# An Enemy of the Bovines Attacks a Boy

**Published:** 1887-08

**Authors:** G. W. Christian

**Affiliations:** Burnet, Texas


### AN ENEMY OF THE BOVINE ATTACKS THE HUMAN FAMILY.

*By G. W. Christian, M. D.. Burnet, Texas.*

[For Daniel's Texas Medical Journal.]

WILLEY McLEAN, a boy of six years, was brought me in the spring of 1886. He was having chills and fever irregularly. Bloated all over, but no ascites. Complexion sallow. Bowels irregular. Appetite capricious, a brown coat on tongue. Was stiff in all his joints and had transient pains, mostly in hip at this time. The pains continued though, and he would cry for hours with pains in different parts of the body. Cholagogue cathartics, diuretics and diaphoretics, tonics and antiperiodics improved the general health, but had no effect upon the pains and stiffness of the joints, nor were alteratives and anti-rheumatics more successful.

The sufferings of the child continued with increasing severity until this spring, when the pains were confined to the back. A

swelling which was taken for a boil appeared and was not differentiated until the appearance of a grub, known here as the Heel Fly. Its exit was speedy followed by perfect health.

Now as to how this enemy gained access into the child, all is conjecture. Possibly eating raw beef containing the embryo. My idea is that he got it in his mouth, by a deposit of eggs having been made on some parts of his body and conveyed to his mouth by his hands. He may have caught the fly and got the eggs on his hands in that way. I think the cow gets the larvae as the horse gets the grubbs; by licking themselves they convey the eggs to the mouth and thence to stomach.

Will some one suggest how to make the diagnosis and treat such cases in the future? This is the third case that has occured within my knowledge.

